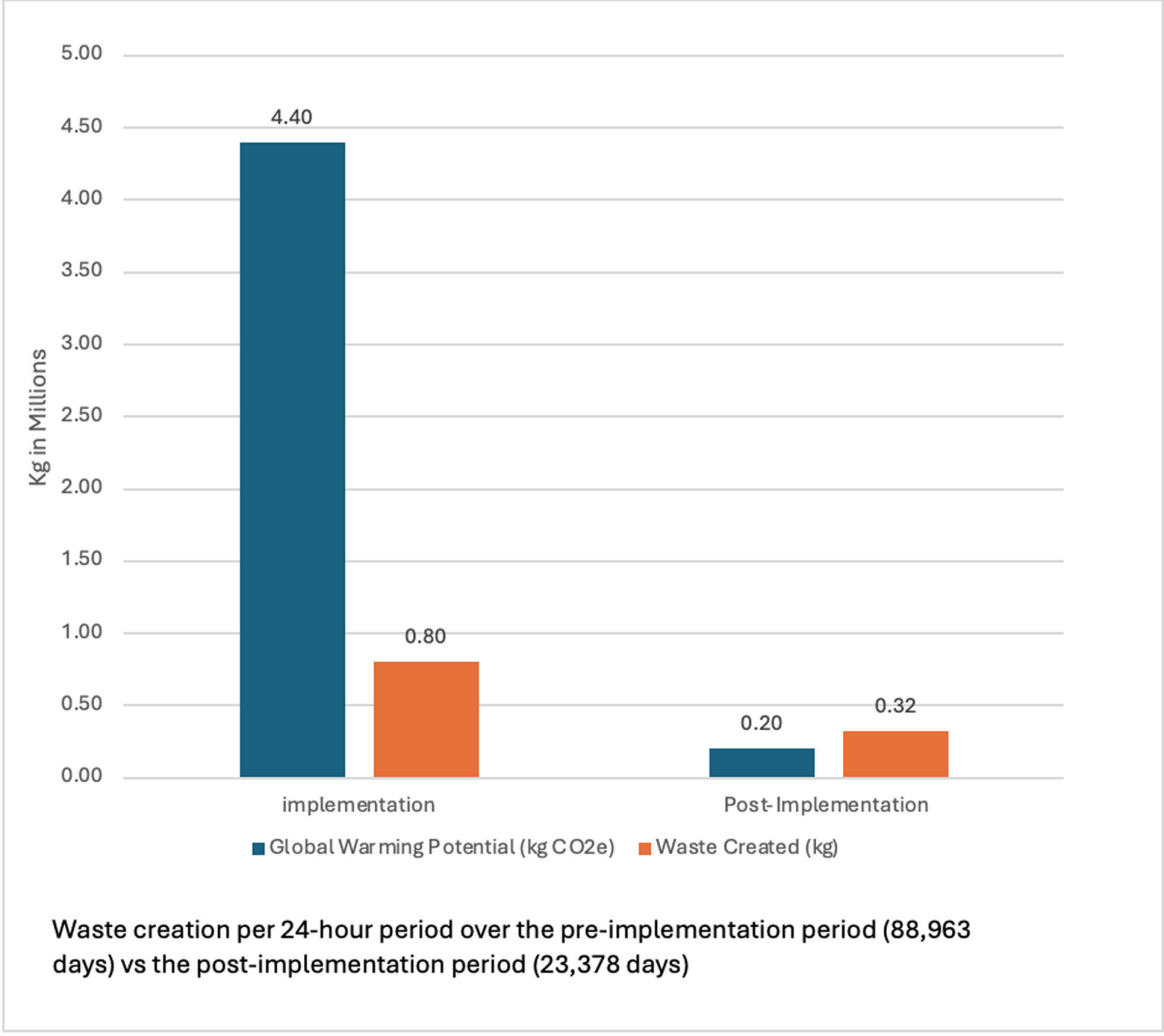# 173 Implementing Admission Screening for Candida auris

**DOI:** 10.1017/ash.2026.10573

**Published:** 2026-06-23

**Authors:** Annie Peacock, Scott Fridkin, Lucy Witt, Jessica Howard-Anderson, Sujit Suchindran, Jesse Jacob, Lindsey Gottlieb, Leila Hojat

**Affiliations:** 1 Emory University; 2 Emory Healthcare and Emory University; 3 Emory University School of Medicine; 4 Emory University Hospital Midtown

## Abstract

**Background:** Contact precautions were used during COVID-19 to reduce nosocomial transmission, though these practices have environmental impacts. We sought to examine the effect of de-implementing contact precautions in a regional healthcare system. **Methods:** We utilized the electronic medical record to identify all hospital-onset COVID-19 cases among hospitalized patients in nine regional hospitals from **Results:** There were 88,963 patient-days of isolation during implementation, and 23,378 patient-days of isolation after implementation. **Conclusions:** Despite de-implementing glove/gown use, we observed no increase in HO COVID-19; however, we benefited from a considerable decrease in both global warming potential and waste.

**Figure:**
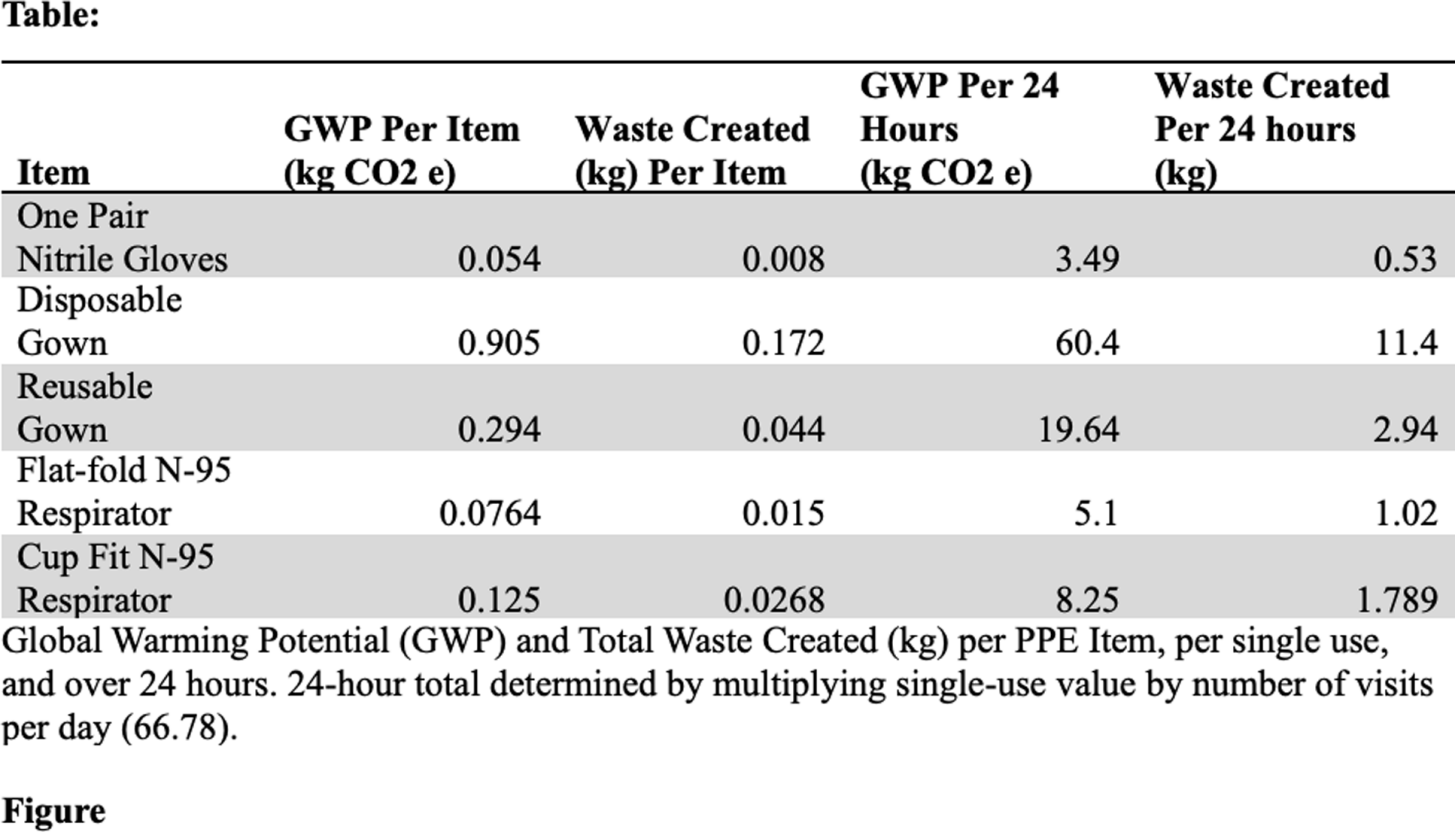

**Figure:**